# Assessment of Dried Plasma Spots (DPS) as an Appropriate Sample Matrix for the Measurement of Circulating Calretinin, a Biomarker for Mesothelioma—A Proof‐Of‐Concept Study

**DOI:** 10.1002/jcla.70271

**Published:** 2026-05-25

**Authors:** Jan Gleichenhagen, Nina Kaiser, Thomas Brüning, Georg Johnen, Daniel G. Weber

**Affiliations:** ^1^ Institute for Prevention and Occupational Medicine of the German Social Accident Insurance, Institute of the Ruhr University Bochum (IPA) Bochum Germany

**Keywords:** asbestos, biomarker, calretinin, cancer, dried blood spots, dried plasma spots, filter cards, marker, mesothelioma

## Abstract

**Background:**

Microsampling using filter cards for dried blood spots (DBS) and dried plasma spots (DPS) is a well‐established technique offering practical advantages for blood collection and subsequent processing. For DBS/DPS, only a small sample volume is required it enables transport and storage at ambient temperature, and it is well suited for decentralized sample collections and resource‐limited settings, as well as collection from elderly individuals, when venous blood collection might be problematic. Many protein‐based biomarker assays like enzyme‐linked immunosorbent assays (ELISA) depend on blood, plasma, or serum as sample matrix. Calretinin is an established biomarker for the early detection of mesothelioma. Herein, we investigated whether DPS are suitable for the determination of calretinin protein by ELISA in comparison to commonly used plasma samples.

**Methods:**

Plasma samples of 47 mesothelioma patients and 47 matched asbestos‐exposed controls were spotted onto filter cards. After drying, calretinin was reconstituted and determined using ELISA.

**Results:**

It was revealed that the use of filter cards, i.e., DPS as sample matrix, was comparable to and in good agreement with the use of plasma. Calretinin could be determined reliably and comparably even after storage for 5 days at ambient temperature. Additionally, the performance of calretinin as biomarker using DPS is improved in comparison to the use of plasma, resulting in a slightly increased sensitivity at constant specificity.

**Conclusion:**

The findings of this proof‐of‐concept study suggest that filter cards represent a valuable alternative sampling method to determine calretinin for the detection or monitoring of mesothelioma.

## Graphical abstract

In this proof‐of‐concept study calretinin as a biomarker for mesothelioma was evaluated using filter cards, i.e., dried plasma spots (DPS) as an alternative sample matrix. After drying, calretinin was reconstituted and determined using ELISA. Calretinin could be reliably determined and the performance of calretinin is slightly improved in comparison to the common use of plasma. The findings suggest that filter cards represent a valuable alternative sampling method to determine calretinin for the detection or monitoring of mesothelioma (Created in BioRender; https://BioRender.com/dcvo6s1).

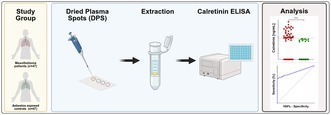

## 1 Introduction

Microsampling using filter cards is a well‐established method [1, 2, 3]. Recent advances in analytical sensitivity have renewed interest in this method, as it enables reliable results from minimal sample volumes. Worldwide, the sampling of dried blood spots (DBS) on filter cards is used in newborn screening programs [4, 5, 6, 7]. DBS offer several practical advantages: they require only small sample volumes, enable ambient temperature transport and storage, and are well suited for decentralized sample collections and resource‐limited settings [1, 8]. In addition, DBS sampling is particularly advantageous for repeated sampling, as it is minimally invasive and well tolerated [9]. The simplicity of blood collection using filter cards is especially relevant in elderly patients, for whom venous access may be limited or unreliable, making capillary‐based collection a practical alternative. Additionally, it might be particularly advantageous for occupational medicine testing when samples are usually taken within companies, which usually have no regular facilities to transport the samples to the appropriate laboratories. While DBS as well as dried plasma spots (DPS) have been widely adopted for small molecule and nucleic acid analysis, their application to peptides and proteins—especially in immunoassays—remains relatively uncommon [1, 8]. Recent studies have demonstrated the feasibility of using DBS/DPS in protein‐based immunoassays, including enzyme‐linked immunosorbent assays (ELISA), with promising results regarding analyte stability and assay performance [10, 11]. However, the application of DBS/DPS for protein‐based cancer diagnostics in clinical studies remains largely exploratory, despite their advantages, e.g., application in areas without close medical supervision and as a self‐sampling tool to monitor diseased individuals.

Mesothelioma is an aggressive cancer of the serous membranes, commonly linked to prior asbestos exposure. The tumor can be classified into three subtypes: epithelioid mesothelioma, which is associated with a more favorable prognosis, sarcomatoid mesothelioma which are linked to poorer outcomes, and biphasic mesothelioma as a mix of both types [12]. Typically, mesothelioma is characterized by a prolonged latency period of up to 50 years; therefore, it is often diagnosed in older individuals. Based on the common diagnosis at late stages, the overall survival (OS), i.e., the period between cancer diagnosis and death, is low, between six and 9 months [12, 13]. Over the past two decades, treatment with pemetrexed and cisplatin was the only approved first‐line therapy that resulted in an increased OS to approximately 12 months after diagnosis [14]. Recent advances in immunotherapy, particularly the use of nivolumab and ipilimumab, have expanded treatment options and extended OS up to 18 months, especially for non‐epithelioid mesothelioma [15].

For a long time, preliminary diagnosis was mostly based on relatively nonspecific symptoms, occupational exposure history and/or incidental CT findings etc. that were then confirmed by biopsy and histopathological examination, including immunohistochemistry [16]. To date, circulating calretinin and mesothelin have been validated in a prospective study for the early detection of mesothelioma, demonstrating a sensitivity of 46% and a specificity of 98% using plasma samples collected up to 15 months prior to the clinical diagnosis [17]. Thus, both protein biomarkers were appropriate for screening in at‐risk populations utilizing regular blood collection procedures, with positive results being an indication for more invasive follow‐up diagnostics. Additionally, calretinin has recently been associated with disease progression in surgically treated mesothelioma patients [18]. As these typically affect elderly people, venous blood sampling can be problematic. The use of microsampling methods, i.e., DBS or DPS, offers a simple method for blood sample collection. Calretinin can be detected in serum, plasma, and pleural effusions of mesothelioma patients [19]. The protein purification method described by Schwaller and colleagues involves denaturing purification followed by simple refolding of the protein through buffer exchange, maintaining its biological functionality and stability [20]. As a result, calretinin exhibits ideal properties for protein recovery from DPS, enabling downstream analysis using ELISA.

The aim of this proof‐of‐concept study was to evaluate the feasibility of DPS as a sample matrix for calretinin determination, serving as an alternative method to provide samples for the detection and monitoring of mesothelioma.

## 2 Material and Methods

### 2.1 Study Group

The study group comprised 47 male mesothelioma patients and 47 male asbestos‐exposed controls. Mesothelioma patients were recruited at the Lungenklinik Heckeshorn, Helios Klinikum Emil von Behring, Berlin, Germany. Matched controls formerly exposed to asbestos were selected from the Molecular Marker (MoMar) cohort [21]. Matching criteria were age and smoking status. Details of the study population are shown in Table 1.

**TABLE 1:** Study group.

	Mesothelioma patients [*n*]	Asbestos‐exposed controls [*n* *]
Total	47	47
Median age (years)	73	73
Age range (years)	40–86	58–93
Ever‐Smoker	32	30
Never‐Smoker	15	15
Histological subtype		
Epithelioid	38	—
Biphasic	5	—
Sarcomatoid	4	—

* Data was missing for one subject, smoking status for another subject.

All participants gave written informed consent. The study was conducted according to the guidelines of the Declaration of Helsinki and approved by the Ethics Committee of the Ruhr University Bochum (reference number 3217–08). Sampling was initiated in December 2008 and concluded in March 2018.

### 2.2 Sample Collection

Blood was collected using EDTA K2 gel monovettes (Sarstedt, Nümbrecht, Germany), which were centrifuged at 2000 x g for 10 min at room temperature. Following the separation of plasma from the cellular fraction by decanting, samples were immediately frozen at −21°C. Samples were transported frozen to the Institute for Prevention and Occupational Medicine of the German Social Accident Insurance, Institute of the Ruhr University Bochum (IPA), thawed at room temperature, aliquoted, and stored at −80°C until use.

### 2.3 Dried Plasma Spots

**FIGURE 1:**
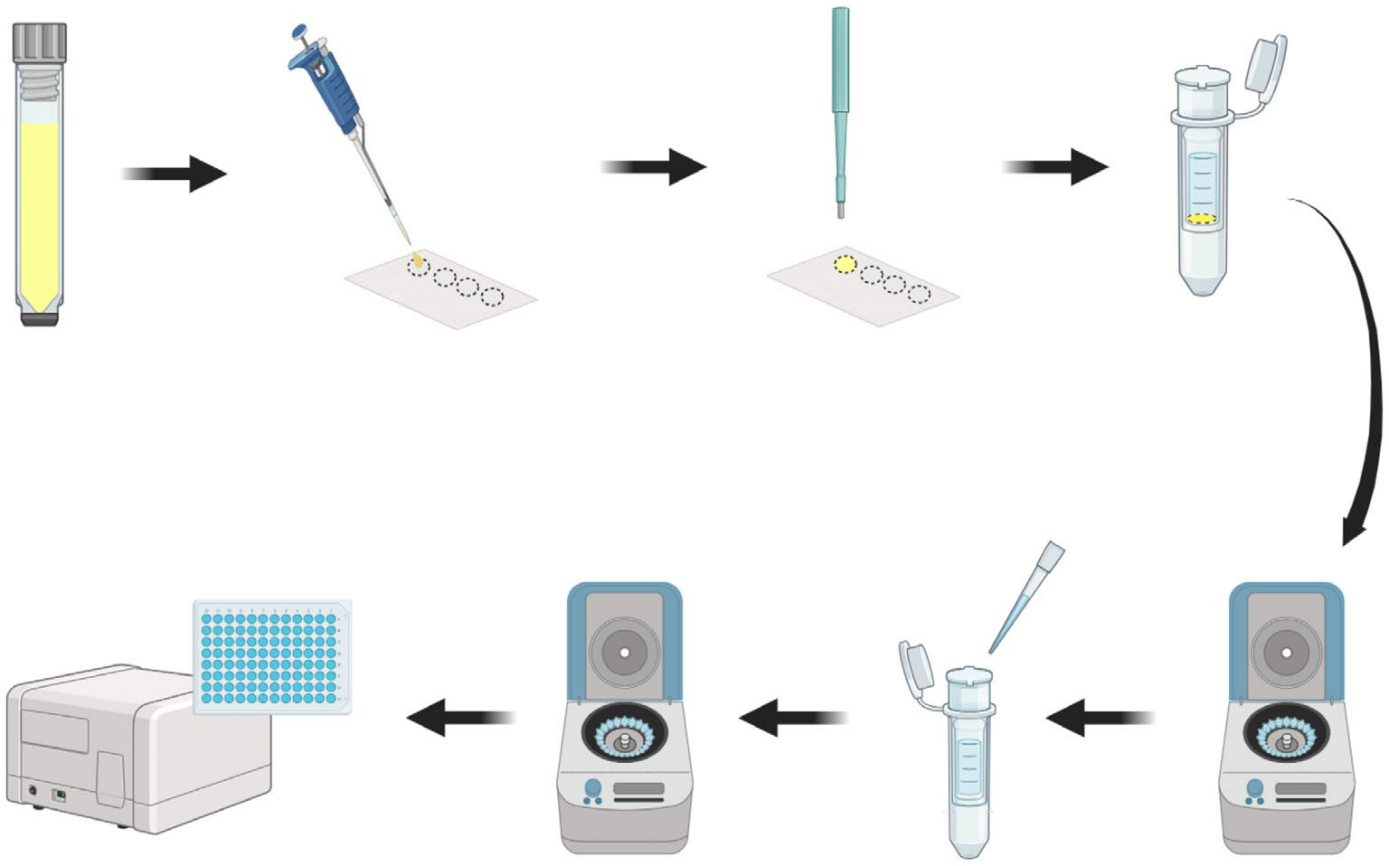
Workflow of dried plasma spots (DPS) handling procedure. Created in BioRender (https://BioRender.com/em86ihz).

The general workflow is depicted in Figure 1. To generate DPS, 30 μL of plasma samples were pipetted onto a single spot of a filter card (Whatman Protein Saver Cards, Merck, Darmstadt, Germany). Unless otherwise mentioned, filter cards were dried for 2 h and then kept overnight at ambient temperature, simulating a shipment by mail from the place of blood collection to the analyzing laboratory. Spots were manually punched out (8 mm diameter) and transferred to a spin cup (Spin Cups—Cellulose Acetate Filter, Thermo Scientific Pierce, Darmstadt, Germany). For resolubilization of dried proteins, the Tris‐based dilution buffer (pH ≈7) from the Calretinin ELISA kit (DLD Diagnostika GmbH, Hamburg, Germany) was used. This buffer contains < 0.05% ProClin and heparin, as well as calcium chloride, which is required to maintain the proper conformation of calretinin necessary for antibody recognition [22]. Initially, 65 μL dilution buffer was pipetted directly onto the punched disc and incubated for 1 h at 37°C with shaking at 1000 rpm in a thermocycler (Eppendorf, Hamburg, Germany). For the first elution step, the spin cup (pore size 0.45 μm) was centrifuged for 2 min at 10,000 x g at room temperature. Subsequently, another 65 μL of dilution buffer were added to the disc and incubated for 10 min at room temperature followed by centrifugation for 2 min at 10,000 x g at room temperature. This workflow resulted in a final volume of 120 μL restored sample solution, achieving a 1:4 dilution relative to the initial sample volume of 30 μL plasma. For determination of calretinin stability using DPS, filter cards were additionally stored for 5 days at ambient temperature, simulating a delay between sampling and further processing. Resolubilization of dried proteins was performed as described above.

### 2.4 Calretinin ELISA Measurements

Concentrations of calretinin in DPS and plasma were determined using the Calretinin ELISA kit (DLD Diagnostika GmbH) according to the manufacturer's instructions. During the procedure, all reagents and samples were equilibrated to 22°C, and incubations were performed at the same temperature. For measurement of calretinin in plasma, samples were diluted 1:5 in the provided dilution buffer, and 50 μL were used for measurement. Following the same procedure, calretinin was determined from extracted DPS using 50 μL of undiluted resolubilized samples. Optical density (OD) was measured on a SpectraMax 384 plus plate reader (Molecular Devices, Sunnyvale, CA, USA). The standard curve was generated by four‐parameter curve fitting using SoftMax Pro 7.0.3 from Molecular Devices (Sunnyvale, CA, USA). All samples were determined in duplicate. The LoD concentration is defined as the value whose mean OD exceeds the mean blank by ≥ 2 standard deviations (SD), operationalized by the lowest analyte calibrator (Cal1 = 0.05 ng/mL), resulting in a LoD of 0.05 ng/mL [22]. The LoQ was defined based on assay precision. A control sample with a concentration close to the clinically relevant cut‐off was measured in duplicate on five independent assay plates. The coefficient of variation calculated from the plate‐wise mean values was approximately 6%. The applied cutoff for DBS and plasma was 0.6 ng/mL, the same as used previously for plasma samples [17]. All measured calretinin concentrations are listed in Table S1.

### 2.5 Statistics/Analysis

Statistical analysis was conducted using GraphPad Prism 10.4.1 (GraphPad Software, Boston, MA, USA). Bland–Altman plots were used to evaluate the effect of the two different sampling methods (DPS and plasma) on the calretinin concentrations, assessing the agreement between the two approaches by comparing the differences with the means of the measurements. Prior to Bland–Altman analysis, calretinin values were log‐transformed to meet the assumption of normal distribution. Wilcoxon matched pairs signed rank test was used to assess statistical significance. Receiver operating characteristics (ROC) curves were used to quantify classification performance. The accuracy of the test was shown by the area under curve (AUC) and the 95% confidence interval (95% CI).

The STARD2015 (An Updated List of Essential Items for Reporting Diagnostic Accuracy Studies) checklist is provided in Table S2 [23].

## 3 Results

### 3.1 Comparing Calretinin Concentrations Using Dried Plasma Spots and Plasma

Using DPS, calretinin was detectable in 47 of 94 samples, showing concentrations above the LoQ (35 mesothelioma and 12 asbestos‐exposed controls). The remaining 47 samples showed no detectable calretinin levels (all< LoD, no values between LoQ and LoD), including 12 mesothelioma and 35 asbestos‐exposed controls. By using DPS, 30 of 47 mesothelioma patients showed calretinin values above the cutoff (≥ 0.6 ng/mL), whereas all control samples remained below the cutoff using DPS, though two showed values near the cutoff (0.51 and 0.57 ng/mL).

Using plasma directly, calretinin was quantifiable (≥ LoQ) in 56 samples (42 mesothelioma patients and 14 asbestos‐exposed controls). Among the remaining 38 samples, 31 exhibited calretinin concentrations between the LoQ and the LoD. Calretinin was undetectable (< LoD) in seven samples, including one from a mesothelioma patient and six from asbestos‐exposed controls. In total, using plasma, 28 mesothelioma patients showed values > 0.6 ng/mL. One control sample was slightly above the cutoff (0.63 ng/mL) and one below (0.56 ng/mL).

**FIGURE 2:**
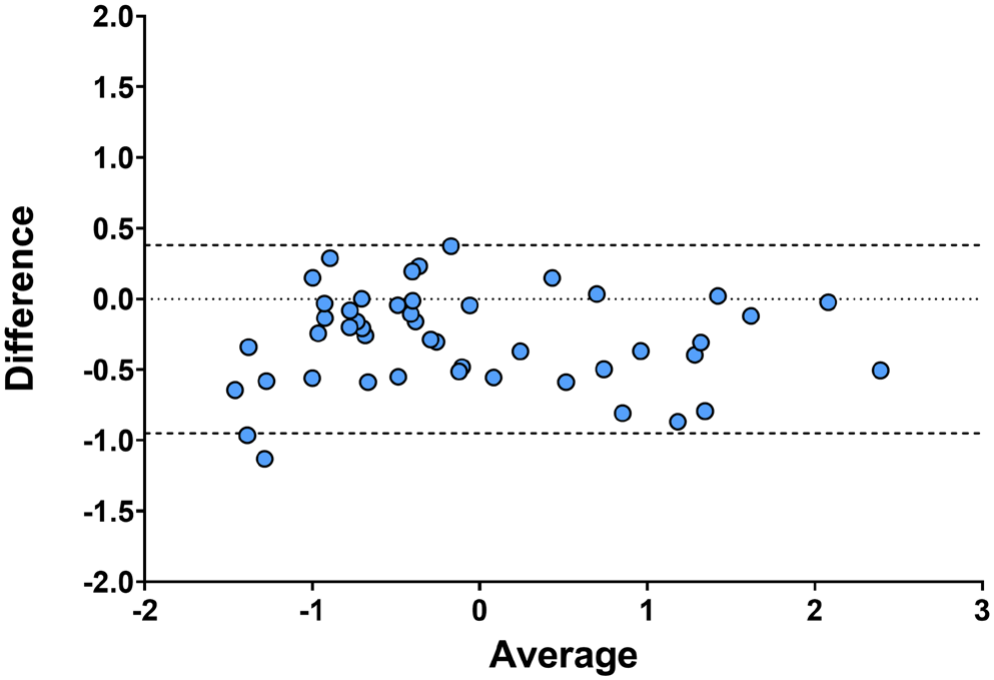
Bland–Altman plot of calretinin concentrations using dried plasma spots (DPS) and plasma as sample matrices. Only values above the limit of detection using dried plasma spots (DPS) and plasma (*n* = 47) were used for analysis. The dotted line represents the median and the dashed lines represent the 95% Limits of Agreement.

To evaluate differences in calretinin determination using DPS and plasma, we performed a Bland–Altman analysis using all samples showing detectable calretinin concentrations (Figure 2). Overall, similar calretinin concentrations could be observed in corresponding DPS and plasma samples, revealing a bias of −0.29, with 95% Limits of Agreement (LoA) ranging from −0.95 to +0.38.

### 3.2 Impact of Storage Time on Calretinin Determination Using DPS

**FIGURE 3:**
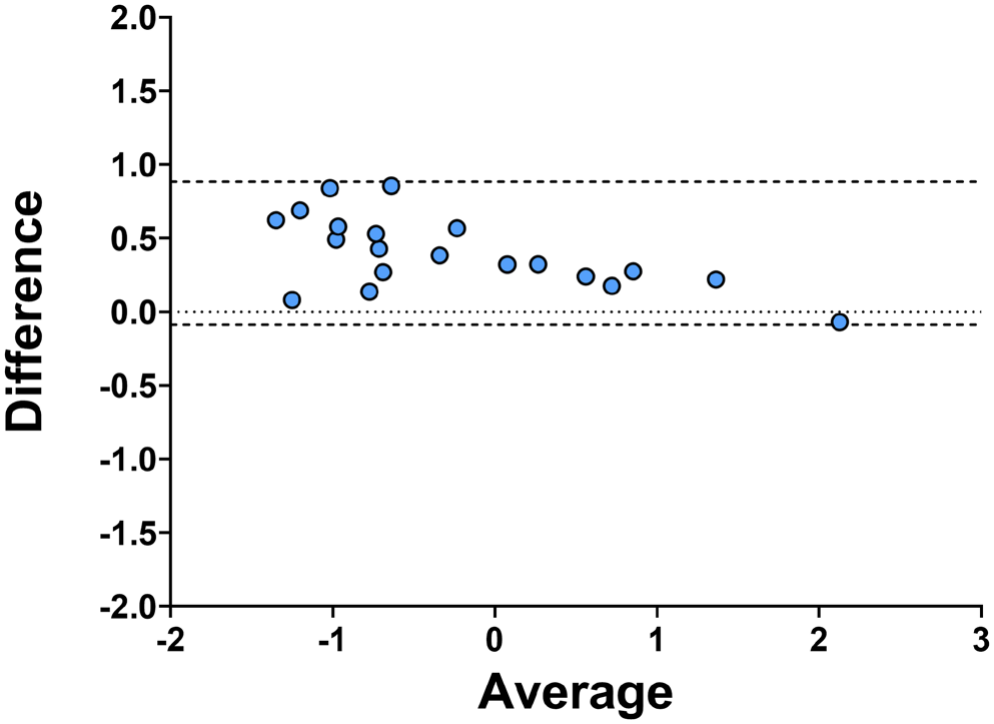
Bland–Altman plot of log‐transformed calretinin concentrations comparing one‐day‐stored and five‐day‐stored dried plasma spots (DPS). Delayed samples were compared to direct processing of DPS (*n* = 22). The dotted line represents the median and the dashed lines represent the Limits of Agreement.

To investigate a potential delay between sampling and processing of the DPS a subset of 40 samples including 20 mesothelioma cases and 20 asbestos‐exposed controls was analyzed. Plasma samples were spotted on filter cards, dried, and left for 5 days at ambient temperature. To assess differences in calretinin determination, Bland–Altman analysis was performed using only samples with detectable calretinin concentrations (*n* = 22). As shown in Figure 3, analysis of one‐day‐stored vs. five‐day‐stored DPS revealed a bias of 0.4 (95% LoA −0.09 to 0.88). Likewise, DPS after 5 days versus plasma showed a bias of 0.2 (95% LoA −0.45 to 0.85) (Figure S1). Of note, using DPS after 5 days at ambient temperature three samples of mesothelioma patients showed calretinin values below the cutoff (0.40 and 0.59 ng/mL) in contrast to the corresponding plasma samples.

### 3.3 Performance of Calretinin as a Biomarker for Mesothelioma Using DPS

**FIGURE 4:**
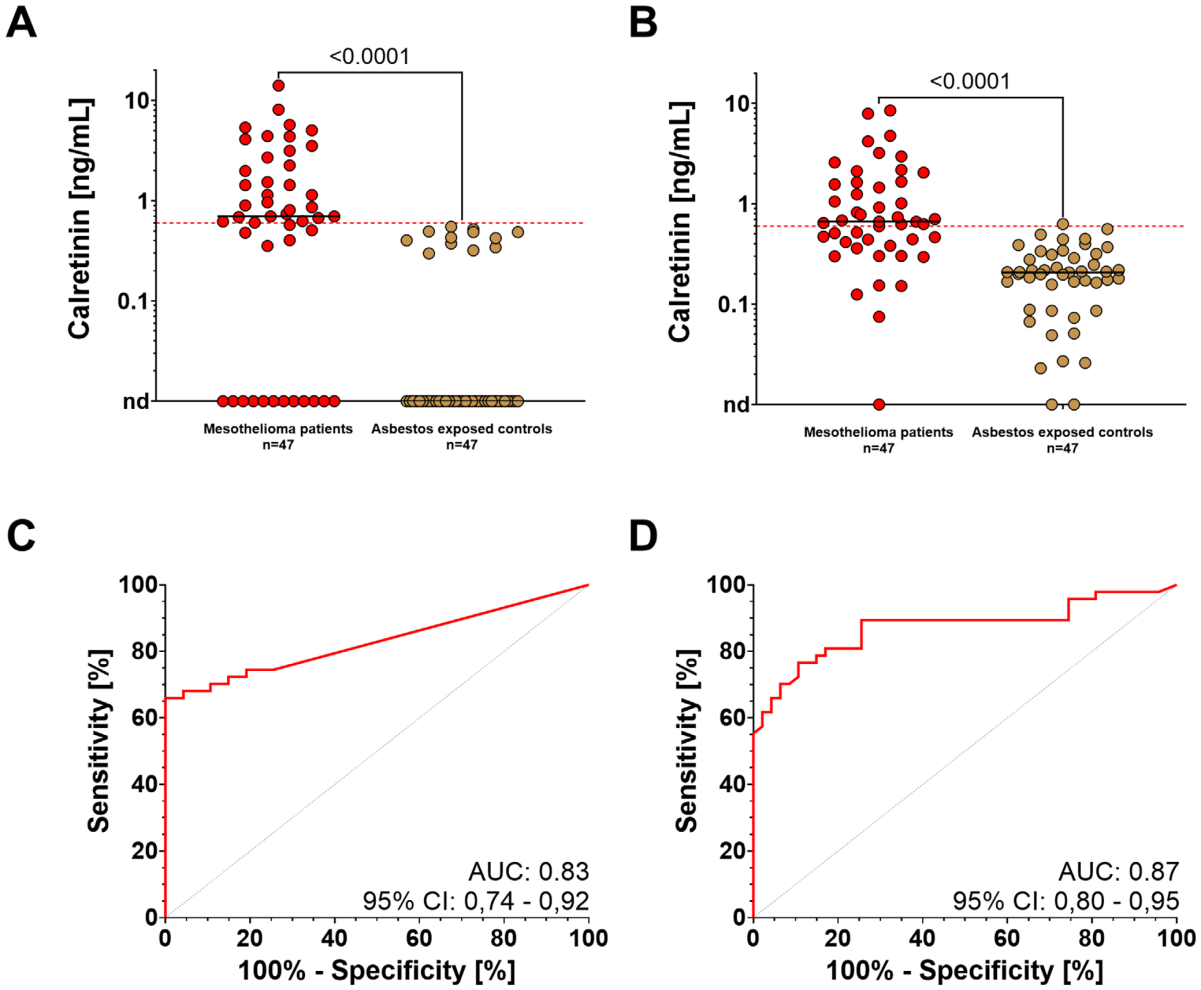
Performance analysis of calretinin using dried plasma spots (DPS). Distribution of the calretinin concentrations in 47 mesothelioma cases and 47 matched asbestos‐exposed controls using DPS (A) and Plasma directly (B). The red dashed line indicates the cutoff value (0.6 ng/mL). Receiver operating characteristic curve based on 47 mesothelioma cases and 47 matched asbestos‐exposed controls using DPS (C) and using Plasma directly (D); (AUC: Area under curve; 95% CI: 95% confidence interval; nd: Not detectable, values below the assay's limit of detection).

The performance of calretinin as a biomarker using DPS and using plasma directly was assessed. Figure 4A,B show the distribution of calretinin concentrations in mesothelioma patients and asbestos‐exposed controls for DPS and plasma, respectively. Using DPS, mesothelioma patients showed higher calretinin concentrations than matched controls, with a median of 0.7 ng/mL (Interquartile range (IQR) 0.01–2.25 ng/mL) and 0.01 ng/mL (IQR 0.01–0.3 ng/mL), respectively. The difference between the groups was statistically significant (*p* < 0.0001). Using plasma directly, the mesothelioma group showed a median of 0.67 ng/mL (IQR 0.01–1.64 ng/mL), while the control group had a median of 0.21 ng/mL (IQR 0.09–0.63 ng/mL).

Figure 4C,D present the results of the ROC analysis, revealing an AUC of 0.83 (95% CI 0.74–0.92) for DPS and 0.87 (95% CI 0.80–0.95) for plasma.

For DPS, using the highest Youden index, an optimal balance between 66% sensitivity and 100% specificity was achieved using a cutoff of 0.56 ng/mL. Applying the cutoff of 0.6 ng/mL resulted in almost the same sensitivity of 64% (95% CI: 52%–78%) and a specificity of 100%.

Using 0.6 ng/mL as cut‐off for calretinin in DPS and plasma resulted in similar true positive, true negative, false positive, and false negative tests (Table 2). Compared to plasma, DPS revealed a slightly increased performance of calretinin (Figure 4C,D).

**TABLE 2:** Calretinin outcome classification represented as confusion matrix of true positive, true negative, false positive, and false negative tests.

	True positive [*n*]	True negative [*n*]	False positive [*n*]	False negative [*n*]
Plasma	29	46	1	18
DPS	30	47	0	17
Detected by				
Plasma and DPS	28	46	0	16
Plasma exclusively	1	0	1	2
DPS exclusively	2	1	0	1

## 4 Discussion

In this study, we demonstrated that DPS is an appropriate sample matrix for the measurement of calretinin. We successfully developed a protocol for recovering calretinin from DPS, resulting in biomarker performance and calretinin values comparable to those obtained using plasma directly.

In our study, we compared DPS and plasma as matrices for the measurement of calretinin by means of identical samples from mesothelioma patients and asbestos‐exposed controls. Calretinin concentrations ranged from 0.001 to 14.080 ng/mL reflecting the full dynamic range of the biomarker expected in real world conditions. The comparison of the two methods demonstrated a consistent detection of calretinin. Notably, higher calretinin values were obtained using DPS at concentrations above 1 ng/mL in comparison to plasma as indicated by the bias of −0.29 in the Bland–Altman analysis. But these differences are considered negligible because the concentrations are well above the applied cutoff of 0.6 ng/mL. Higher calretinin concentrations have currently no further clinical implications because no association between calretinin concentration and TNM staging and tumor grading has been observed yet [22]. The reproducibility of calretinin determination and sample stability were confirmed using DPS samples stored for 5 days at ambient temperature, simulating a prolonged time between sample collection and processing. Even after 5 days' storage of the DPS at ambient temperature, measured calretinin concentrations remained comparable to the plasma‐based values. However, calretinin values of three samples from mesothelioma patients dropped below the cutoff. Notably, two of these samples exhibited concentrations close to the cutoff. Thus, an adjustment of the cutoff value might be required when switching the sample matrix from plasma to DPS and longer storage times [10]. ROC analysis and Youden index indicated that the optimal cutoff for calretinin measurement in DPS would be 0.56 ng/mL, a little bit lower than the currently used cut‐off of 0.6 ng/mL [17]. However, using 0.56 ng/mL as cut‐off did not improve the performance of the biomarker in this study.

Based on the cut‐off of 0.6 ng/mL, the sensitivity of 64% and specificity of 100% obtained using DPS are in good agreement with previous studies using the plasma‐based approach. Johnen et al. initially showed that calretinin had a sensitivity of 71% at a predefined specificity of 95% for the differentiation between mesothelioma patients and asbestos‐exposed controls using the original in‐house calretinin assay [24]. Zupanc et al. revealed a sensitivity of 45% and a specificity of 99% [25] and Jimenez‐Ramirez et al. reported a sensitivity of 81% in males and 68% in females, both at a specificity of 95% [26]. Thus, using the DPS method, the observed performance of calretinin as a biomarker for mesothelioma is in line with previous results using plasma directly. However, sensitivity seems to be slightly higher using DPS compared to plasma. ROC analysis revealed a sensitivity of 64% and 62% for DPS and plasma, respectively. Notably, the observed difference in sensitivity was driven by only two samples and therefore may be attributed to the small sample size in this initial proof‐of‐concept study. Importantly, negative results in the control group were consistently confirmed, with 35 out of 47 samples from asbestos‐exposed subjects showing calretinin values below the LoD. This emphasizes the high specificity obtained with the DPS method that is in accordance with the direct measurement in plasma.

As DPS is not equivalent to plasma, it represents a unique sample type encompassing its own strengths and weaknesses [1]. The drying process may influence the stability, solubility, and biological activity of proteins and other analytes present in the sample. One possible explanation for the slightly increased specificity compared to the conventional plasma‐based approach might be that nonspecific interactions, caused by partially denatured or aggregated plasma proteins, may be mitigated by the DPS matrix. Protein aggregation can occur under certain conditions, such as high temperatures, changes in pH or salt concentration, but also at low temperatures [27]. Physical instability of proteins can include secondary or tertiary structural changes, which can expose hydrophobic parts of proteins previously buried within the fold [28, 29]. Aggregates with a higher molecular mass might just become stuck in the pores of the filter card membrane, whereas other denatured proteins may form stronger bonds by hydrophobic forces. We hypothesize that such aggregates, if present in liquid plasma, may interfere with immunoassays like ELISA by contributing to nonspecific binding [30]. A well‐known example is the interference of Rheumatoid factor in immunoassays [31, 32, 33, 34]. In contrast, these aggregates or interferences are likely to be retained on the DPS filter matrix due to limited re‐solubilization, thereby preventing their interference in the assay and reducing the likelihood of false positive results. This may also explain why most control samples were below the LoD using DPS. In contrast, calretinin concentrations in plasma were detectable in nearly all samples, suggesting generally higher calretinin levels using this matrix. Thus, DPS—as a sample matrix for the detection of calretinin by ELISA—appears to be a valid alternative, offering promising performance‐related benefits besides the fundamental advantages regarding sampling, shipment, and storage.

A limitation of this study is the relatively small sample size of the analyzed group. Thus, to verify our proof‐of‐concept findings, larger studies are needed in the future. Additionally, the MoMar cohort could be used to explore the potential of calretinin extracted from filter cards for the early detection of mesothelioma [21]. Furthermore, our current approach still requires centrifugation of whole blood to obtain plasma, whereas the direct use of DBS would represent the most desirable and streamlined workflow at present. DBS was successfully implemented in the determination of inflammatory cytokines even for a multiplex immunoassay. McDade and his team were able to quantify the cytokines IL6, IL10, and TNFα in DBS with acceptable levels of precision and reliability [35]. Additionally, Beyerl and colleagues showed that DBS could also be used for SARS‐CoV‐2 serology [36]. Thus, one of the next steps will be the evaluation of calretinin measurement using DBS, because DBS enables a simplified sample collection. In principle, calretinin can be determined in whole blood (data not shown). Therefore, this approach not only serves as a promising alternative for detection of mesothelioma, but also for monitoring of mesothelioma patients. DBS can enable repeated sampling at shorter intervals even through home‐based collection by patients themselves. Moreover, DBS requires less blood and offers a minimally invasive finger‐prick procedure, improving acceptance among elderly individuals with limited tolerance for venous blood sampling. However, DBS also has some challenges that must be addressed, including hematocrit variability, blood viscosity, and other pre‐analytical factors discussed previously [1, 8, 37]. Regarding this, recent advances have allowed the direct retrieval of plasma from whole blood using filter cards equipped with an integrated membrane that facilitates plasma separation, representing a promising alternative method [2, 38, 39].

## 5 Conclusion

This proof‐of‐concept study demonstrated that DPS is a reliable sample matrix for the determination of calretinin. These findings may serve as a foundation for future developments toward simplified and user‐friendly sample collection procedures applicable for the detection and monitoring of mesothelioma. Additionally, DPS could be suitable for use in areas without close medical facilities as well as resource‐limited and decentralized healthcare environments. This is of actual relevance for programs for early detection of mesothelioma like “EVA‐Mesothel” that were recently implemented in Germany as a nationwide offer for workers previously occupationally exposed to asbestos [40].

## Author Contributions

J.G.: Conceived and designed the experiments, developed the workflow, supervised experimental procedures, performed data analysis, and drafted the manuscript. N.K.: Conducted measurements, coordinated sample logistics, and contributed to sample selection. T.B.: Supervised the study design. G.J.: Supervised and contributed to the study design and critically reviewed the manuscript. D.G.W.: Contributed to the study design, supervised workflow development, performed data analysis, and helped to draft the manuscript. All authors read and approved the manuscript.

## Funding

The authors have nothing to report.

## Ethics Statement

By the Ethics Committee of the Ruhr University Bochum (reference number 3217–08) (page 5, line 96).

## Consent

All participants gave written informed consent (page 5, line 95).

## Conflicts of Interest

The authors are employees of the Institute for Prevention and Occupational Medicine of the German Social Accident Insurance, Institute of the Ruhr University Bochum (IPA). The authors are independent from the German Social Accident Insurance (DGUV) in study design, access to the collected data, responsibility for data analysis and interpretation, and the right to publish. The views expressed in this paper are those of the authors and not necessarily those of the DGUV. The IPA has supplied DLD Diagnostika GmbH with the antibodies to produce the Calretinin ELISA kits. In turn, the IPA has received Calretinin ELISA kits at a reduced price and may benefit from future sales of the kits.

## Supporting information


**Table S1:** Measured calretinin concentrations. Plasma: Calretinin measurement using plasma directly. DPS: Calretinin measurement using dried plasma spots next day. DPS‐5d: Calretinin using dried plasma spots left for 5 days at ambient temperature. n.d.: not detectable.
**Table S2:** STARD 2015 Checklist.
**Figure S1:** Bland–Altman plot comparing direct use of plasma vs. five‐day‐storage at ambient temperature of dried plasma spots (DPS). Dotted line represents the median and dashed lines the Limits of Agreement.

## Data Availability

The data that supports the findings of this study are available in the Supporting Information of this article.
