# Supplementary material for: Assessment of Dried Plasma Spots (DPS) as an Appropriate Sample Matrix for the Measurement of Circulating Calretinin, a Biomarker for Mesothelioma—A Proof‐Of‐Concept Study
Source: J Clin Lab Anal. 2026 May 25;40(11):e70271. doi: 10.1002/jcla.70271 (PMC13267156; doi:10.1002/jcla.70271)

Assessment of Dried Plasma Spots (DPS) as an Appropriate Sample Matrix for the Measurement of Circulating Calretinin, a Biomarker for Mesothelioma – A proof-of-concept study

Jan Gleichenhagen*, Nina Kaiser, Thomas Brüning, Georg Johnen, and Daniel G. Weber

Institute for Prevention and Occupational Medicine of the German Social Accident Insurance, Institute of the Ruhr University Bochum (IPA), Bochum, Germany

# Supporting information

**Table S1: Measured calretinin concentrations.** Plasma: Calretinin measurement using plasma directly. DPS: Calretinin measurement using dried plasma spots next day. DPS-5d: Calretinin using dried plasma spots left for 5 days at ambient temperature. n.d.: not detectable

| SampleType | Plasma | DPS | DPS-5d |
| --- | --- | --- | --- |
| Mesothelioma | 0.822 | 1.433 |  |
| Mesothelioma | 1.564 | 3.513 |  |
| Mesothelioma | 1.057 | 1.533 | 1.111 |
| Mesothelioma | 0.628 | 0.698 |  |
| Mesothelioma | 0.646 | 0.861 | 0.587 |
| Mesothelioma | 0.303 | n.d. |  |
| Mesothelioma | 0.361 | n.d. |  |
| Mesothelioma | 2.11 | 5.031 |  |
| Mesothelioma | 2.959 | 4.4 |  |
| Mesothelioma | 0.382 | 0.688 |  |
| Mesothelioma | 0.663 | 0.898 |  |
| Mesothelioma | 1.659 | 1.431 |  |
| Mesothelioma | 7.909 | 8.093 | 8.68 |
| Mesothelioma | 0.51 | n.d. |  |
| Mesothelioma | 0.152 | n.d. |  |
| Mesothelioma | 0.599 | 0.626 | 0.369 |
| Mesothelioma | 0.416 | 0.508 | 0.285 |
| Mesothelioma | 0.125 | n.d. | n.d. |
| Mesothelioma | 0.442 | 0.48 | 0.294 |
| Mesothelioma | 0.683 | 1.142 |  |
| Mesothelioma | 0.443 | 0.574 | 0.439 |
| Mesothelioma | 0.472 | 0.354 | 0.19 |
| Mesothelioma | 2.579 | 5.702 |  |
| Mesothelioma | 2.043 | 1.976 | 1.554 |
| Mesothelioma | 1.247 | 2.246 | 1.885 |
| Mesothelioma | 3.207 | 4.363 | 3.503 |
| Mesothelioma | 0.629 | 0.738 |  |
| Mesothelioma | 0.075 | n.d. |  |
| Mesothelioma | 8.49 | 14.08 |  |
| Mesothelioma | 0.005 | n.d. |  |
| Mesothelioma | 1.635 | 2.689 | 2.045 |
| Mesothelioma | 2.172 | 3.142 |  |
| Mesothelioma | 0.466 | 0.809 | 0.344 |
| Mesothelioma | 1.454 | n.d. |  |
| Mesothelioma | 0.154 | 0.404 |  |
| Mesothelioma | 0.706 | 1.144 |  |
| Mesothelioma | 0.92 | 0.963 |  |
| Mesothelioma | 4.75 | 5.361 |  |
| Mesothelioma | 0.302 | n.d. | n.d. |
| Mesothelioma | 0.3 | n.d. |  |
| Mesothelioma | 0.665 | 0.674 |  |
| Mesothelioma | 1.014 | 0.698 |  |
| Mesothelioma | 0.736 | 0.606 | 0.395 |
| Mesothelioma | 4.18 | 4.099 |  |
| Mesothelioma | 0.781 | 0.62 |  |
| Mesothelioma | 0.519 | n.d. |  |
| Mesothelioma | 0.296 | n.d. |  |
| Asbestos exposed control | 0.443 | 0.52 |  |
| Asbestos exposed control | 0.231 | n.d. |  |
| Asbestos exposed control | 0.164 | n.d. |  |
| Asbestos exposed control | 0.397 | 0.342 |  |
| Asbestos exposed control | 0.172 | n.d. |  |
| Asbestos exposed control | 0.247 | n.d. |  |
| Asbestos exposed control | 0.218 | n.d. | 0.198 |
| Asbestos exposed control | 0.086 | n.d. |  |
| Asbestos exposed control | 0.049 | n.d. |  |
| Asbestos exposed control | 0.157 | 0.487 |  |
| Asbestos exposed control | 0.217 | n.d. |  |
| Asbestos exposed control | 0.2 | n.d. |  |
| Asbestos exposed control | 0.37 | 0.424 | 0.213 |
| Asbestos exposed control | 0.185 | n.d. |  |
| Asbestos exposed control | 0.051 | n.d. |  |
| Asbestos exposed control | 0.199 | n.d. | n.d. |
| Asbestos exposed control | 0.288 | n.d. |  |
| Asbestos exposed control | 0.026 | n.d. |  |
| Asbestos exposed control | 0.086 | n.d. |  |
| Asbestos exposed control | 0.207 | n.d. |  |
| Asbestos exposed control | 0.561 | n.d. |  |
| Asbestos exposed control | 0.067 | n.d. | n.d. |
| Asbestos exposed control | 0.447 | 0.55 | 0.238 |
| Asbestos exposed control | 0.168 | 0.32 |  |
| Asbestos exposed control | 0.18 | n.d. |  |
| Asbestos exposed control | 0.088 | n.d. | n.d. |
| Asbestos exposed control | 0.168 | n.d. | n.d. |
| Asbestos exposed control | 0.175 | n.d. | n.d. |
| Asbestos exposed control | 0.209 | 0.374 |  |
| Asbestos exposed control | 0.343 | n.d. |  |
| Asbestos exposed control | 0.005 | n.d. | n.d. |
| Asbestos exposed control | 0.389 | 0.402 |  |
| Asbestos exposed control | 0.337 | 0.43 |  |
| Asbestos exposed control | 0.209 | n.d. | 0.218 |
| Asbestos exposed control | 0.005 | n.d. | n.d. |
| Asbestos exposed control | 0.311 | n.d. | n.d. |
| Asbestos exposed control | 0.278 | 0.487 |  |
| Asbestos exposed control | 0.629 | n.d. |  |
| Asbestos exposed control | 0.219 | n.d. |  |
| Asbestos exposed control | 0.197 | n.d. | n.d. |
| Asbestos exposed control | 0.318 | n.d. |  |
| Asbestos exposed control | 0.027 | n.d. |  |
| Asbestos exposed control | 0.211 | n.d. | n.d. |
| Asbestos exposed control | 0.023 | n.d. |  |
| Asbestos exposed control | 0.212 | 0.298 | 0.275 |
| Asbestos exposed control | 0.494 | 0.494 | 0.431 |
| Asbestos exposed control | 0.073 | n.d. | n.d. |

**Table S2: STARD 2015 Checklist**

|  | **Section & Topic** | **No** | **Item** | **Reported on page #** |
| --- | --- | --- | --- | --- |
|  |  |  |  |  |
|  | **TITLE OR ABSTRACT** |  |  |  |
|  |  | **1** | Identification as a study of diagnostic accuracy using at least one measure of accuracy  (such as sensitivity, specificity, predictive values, or AUC) | 2 |
|  | **ABSTRACT** |  |  |  |
|  |  | **2** | Structured summary of study design, methods, results, and conclusions  (for specific guidance, see STARD for Abstracts) | 2 |
|  | **INTRODUCTION** |  |  |  |
|  |  | **3** | Scientific and clinical background, including the intended use and clinical role of the index test | 3-4 |
|  |  | **4** | Study objectives and hypotheses | 4 |
|  | **METHODS** |  |  |  |
|  | *Study design* | **5** | Whether data collection was planned before the index test and reference standard  were performed (prospective study) or after (retrospective study) | 4-5 |
|  | *Participants* | **6** | Eligibility criteria | 5 |
|  |  | **7** | On what basis potentially eligible participants were identified  (such as symptoms, results from previous tests, inclusion in registry) | 5 |
|  |  | **8** | Where and when potentially eligible participants were identified (setting, location and dates) | 4 |
|  |  | **9** | Whether participants formed a consecutive, random or convenience series | na |
|  | *Test methods* | **10a** | Index test, in sufficient detail to allow replication | 6 |
|  |  | **10b** | Reference standard, in sufficient detail to allow replication | 7 |
|  |  | **11** | Rationale for choosing the reference standard (if alternatives exist) | 4 |
|  |  | **12a** | Definition of and rationale for test positivity cut-offs or result categories  of the index test, distinguishing pre-specified from exploratory | 7, 13 |
|  |  | **12b** | Definition of and rationale for test positivity cut-offs or result categories  of the reference standard, distinguishing pre-specified from exploratory | 7 |
|  |  | **13a** | Whether clinical information and reference standard results were available  to the performers/readers of the index test | 4-5 |
|  |  | **13b** | Whether clinical information and index test results were available  to the assessors of the reference standard | 4-5 |
|  | *Analysis* | **14** | Methods for estimating or comparing measures of diagnostic accuracy | 6-8 |
|  |  | **15** | How indeterminate index test or reference standard results were handled | na |
|  |  | **16** | How missing data on the index test and reference standard were handled | na |
|  |  | **17** | Any analyses of variability in diagnostic accuracy, distinguishing pre-specified from exploratory | na |
|  |  | **18** | Intended sample size and how it was determined | 5 |
|  | **RESULTS** |  |  |  |
|  | *Participants* | **19** | Flow of participants, using a diagram | na |
|  |  | **20** | Baseline demographic and clinical characteristics of participants | 5 |
|  |  | **21a** | Distribution of severity of disease in those with the target condition | 5 |
|  |  | **21b** | Distribution of alternative diagnoses in those without the target condition | 5 |
|  |  | **22** | Time interval and any clinical interventions between index test and reference standard | na |
|  | *Test results* | **23** | Cross tabulation of the index test results (or their distribution)  by the results of the reference standard | 12 |
|  |  | **24** | Estimates of diagnostic accuracy and their precision (such as 95% confidence intervals) | 8-11 |
|  |  | **25** | Any adverse events from performing the index test or the reference standard | 12 |
|  | **DISCUSSION** |  |  |  |
|  |  | **26** | Study limitations, including sources of potential bias, statistical uncertainty, and generalisability | 14 |
|  |  | **27** | Implications for practice, including the intended use and clinical role of the index test | 15 |
|  | **OTHER INFORMATION** |  |  |  |
|  |  | **28** | Registration number and name of registry | 5 |
|  |  | **29** | Where the full study protocol can be accessed | na |
|  |  | **30** | Sources of funding and other support; role of funders | Conflict of Interest statement |
|  |  |  |  |  |

**Figure S1: Bland-Altman plot comparing direct use of plasma vs. five-day-storage at ambient temperature of dried plasma spots (DPS)**. Dotted line represents the median and dashed lines the Limits of Agreement.


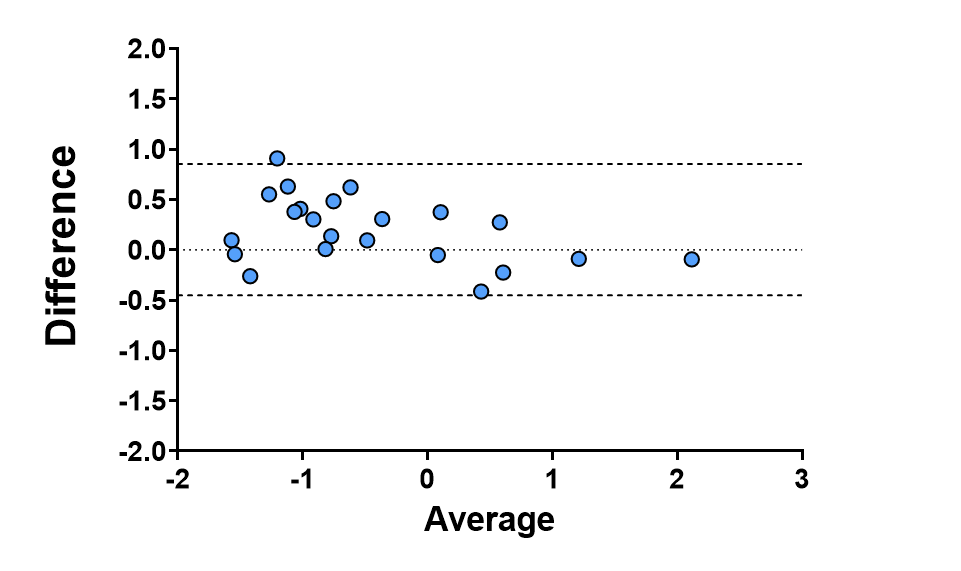

Supplement: Supplementary file 1 — Table S1: Measured calretinin concentrations. Plasma: Calretinin measurement using plasma directly. DPS: Calretinin measurement using dried plasma spots next day. DPS‐5d: Calretinin using dried plasma spots left for 5 days at ambient temperature. n.d.: not detectable. Table S2: STARD 2015 Checklist. Figure S1: Bland–Altman plot comparing direct use of plasma vs. five‐day‐storage at ambient temperature of dried plasma spots (DPS). Dotted line represents the median and dashed lines the Limits of Agreement. [file JCLA-40-e70271-s001.docx]
